# Assessing the Utility of a Patient-Facing Diagnostic Tool Among Individuals With Hypermobile Ehlers-Danlos Syndrome: Focus Group Study

**DOI:** 10.2196/49720

**Published:** 2024-09-26

**Authors:** Jessica Goehringer, Abigail Kosmin, Natalie Laible, Katrina Romagnoli

**Affiliations:** 1 Department of Genomic Health Geisinger Danville, PA United States; 2 Joan H. Marks Graduate Program in Human Genetics Sarah Lawrence College Bronxville, NY United States; 3 Magee-Womens Hospital Pittsburgh, PA United States; 4 GeneScreen Counseling Bernardsville, NJ United States; 5 Department of Population Health Sciences Geisinger Danville, PA United States

**Keywords:** diagnostic tool, hypermobile Ehlers-Danlos syndrome, patient experiences, diagnostic odyssey, affinity mapping, mobile health app, mobile phone

## Abstract

**Background:**

Hypermobile Ehlers-Danlos syndrome (hEDS), characterized by joint hypermobility, skin laxity, and tissue fragility, is thought to be the most common inherited connective tissue disorder, with millions affected worldwide. Diagnosing this condition remains a challenge that can impact quality of life for individuals with hEDS. Many with hEDS describe extended diagnostic odysseys involving exorbitant time and monetary investment. This delay is due to the complexity of diagnosis, symptom overlap with other conditions, and limited access to providers. Many primary care providers are unfamiliar with hEDS, compounded by genetics clinics that do not accept referrals for hEDS evaluation and long waits for genetics clinics that do evaluate for hEDS, leaving patients without sufficient options.

**Objective:**

This study explored the user experience, quality, and utility of a prototype of a patient-facing diagnostic tool intended to support clinician diagnosis for individuals with symptoms of hEDS. The questions included within the prototype are aligned with the 2017 international classification of Ehlers-Danlos syndromes. This study explored how this tool may help patients communicate information about hEDS to their physicians, influencing the diagnosis of hEDS and affecting patient experience.

**Methods:**

Participants clinically diagnosed with hEDS were recruited from either a medical center or private groups on a social media platform. Interested participants provided verbal consent, completed questionnaires about their diagnosis, and were invited to join an internet-based focus group to share their thoughts and opinions on a diagnostic tool prototype. Participants were invited to complete the Mobile App Rating Scale (MARS) to evaluate their experience viewing the diagnostic tool. The MARS is a framework for evaluating mobile health apps across 4 dimensions: engagement, functionality, esthetics, and information quality. Qualitative data were analyzed using affinity mapping to organize information and inductively create themes that were categorized within the MARS framework dimensions to help identify strengths and weaknesses of the diagnostic tool prototype.

**Results:**

In total, 15 individuals participated in the internet-based focus groups; 3 (20%) completed the MARS. Through affinity diagramming, 2 main categories of responses were identified, including responses related to the user interface and responses related to the application of the tool. Each category included several themes and subthemes that mapped well to the 4 MARS dimensions. The analysis showed that the tool held value and utility among the participants diagnosed with hEDS. The shareable ending summary sheet provided by the tool stood out as a strength for facilitating communication between patient and provider during the diagnostic evaluation.

**Conclusions:**

The results provide insights on the perceived utility and value of the tool, including preferred phrasing, layout and design preferences, and tool accessibility. The participants expressed that the tool may improve the hEDS diagnostic odyssey and help educate providers about the diagnostic process.

## Introduction

### Background

Ehlers-Danlos syndromes (EDSs) are a group of inherited disorders of connective tissue that result from impaired collagen synthesis throughout the body [1]. Although phenotypically and genetically distinct, the 13 subtypes share features including varying levels of hyperextensible skin, hypermobile joints, and tissue fragility that can manifest as easy bruising, delayed healing, and atrophic scarring [2]. Various body systems, tissues, and organs may be involved depending on the subtype and influence severity. At the most severe end of the spectrum, vascular EDS carries a risk of spontaneous arterial rupture and sudden death. This risk is not typically associated with the most common and less severe (by comparison) hypermobile EDS (hEDS) type. hEDS is an autosomal dominant condition and generally has less frequent cardiac and skin manifestations than the other subtypes. It is characterized by frequent joint subluxations and dislocations due to joint hypermobility, hyperextensible skin, easy bruising, chronic musculoskeletal pain, and a myriad of associated comorbidities (eg, migraine, chronic fatigue, gut dysmotility, small fiber neuropathy, postural orthostatic tachycardia syndrome, and anxiety) that contribute to significant effects on quality of life and daily functioning [3]. It is currently the only form of EDS without a known genetic etiology, necessitating that diagnosis be made via a detailed clinical examination.

Descriptions of manifestations of EDS, including lax joints and skin findings, date back hundreds of years [4]. However, classification of EDS as a distinct condition was described during the first decade of the 20th century by Edvard Ehlers and Henri Danlos [5]. The classification of the different subtypes has changed several times since, speaking to the complexity of this group of disorders. The Berlin nosology classified 11 EDS subtypes based on clinical symptoms and inheritance pattern [6]. The Villefranche nosology classified 6 subtypes using knowledge of the molecular and biochemical underpinnings [7]. The current nosology, which classifies 13 EDS subtypes and defines major and minor criteria for each, was proposed by the International Consortium on EDS in 2017 and published in the *American Journal of Medical Genetics Part C: Seminars in Medical Genetics* [2]*.* These criteria are more stringent than previous criteria, an effort to improve the definition of the subtypes and lessen misdiagnoses [8].

Historically, hEDS has been considered a rare condition, but there is more recent evidence suggesting that it is substantially more prevalent [9]. With an estimated 255 million affected worldwide [10], hEDS is the most common connective tissue disorder and, by extension, the most common EDS subtype [11]. However, the condition often goes undiagnosed for years, with an average period of 14 years between onset of symptoms and diagnosis [12]. A recent study presented at the 2021 National Society of Genetic Counselors annual conference researched the diagnostic odyssey of patients with joint hypermobility and found that, on average, participants with self-reported joint hypermobility or hEDS saw 6 different providers over multiple years when trying to be accurately diagnosed [13]. Diagnostic delays exceeding 20 years have been reported for patients with hEDS [14,15]. Delayed diagnosis can lead to excessive time and financial investment, redundant investigations, incorrect therapies (that may pose risk to patients with hypermobility), delay of appropriate treatment, and progression of the disease [16,17].

### Barriers to Diagnosis

In addition to the inherent complexity of diagnosing a disorder with multisystem involvement and nonspecific symptoms (eg, chronic pain and fatigue), there are provider-related barriers to diagnosis for individuals with hEDS [18]. Patients are most often referred to medical geneticists for evaluation of hEDS in the United States. Despite the expertise of genetics professionals, it is not clear whether this is the correct medical home for those with hEDS, prompting other models to be considered [19]. This, in part, may be due to the relative scarcity of genetics appointments available in relation to the quantity of referrals for hEDS [13]. The American College of Medical Genetics and Genomics conducted a needs assessment in 2015 to determine whether the United States will have a sufficient workforce of qualified health professionals to meet the genetic health care needs of the population in the future [20]. The American College of Medical Genetics and Genomics estimates that the United States has 1 full-time equivalent medical geneticist per 600,000 people, which is 2.5 times lower than the estimated need [21]. A similar struggle exists in parts of Europe, where there is a wide disparity of staffing levels in clinical genetics units [22].

Limited accessibility to genetics specialists has, in some cases, resulted in genetics clinics declining to see patients referred for an hEDS evaluation [19]. Anecdotally, the authors have been a part of discussions with several genetic counselors seeking advice on where to send patients in need of evaluation for and proper management of hEDS. It has been a topic discussed at national genetics conferences [23] and on professional discussion boards. The genetics specialty clinics that accept patient referrals for hEDS often have patient waitlists exceeding a 2- to 3-year wait time. Some genetics clinics accepting hEDS referrals have or are considering other models because they cannot keep up with demand [19]. Some of these models include referring the patient back to their general practitioner with resources to support the diagnosis and management of EDS, putting age restrictions on referrals, and limiting the number of referrals accepted in a given period [19].

Consultations with and referral to providers inexperienced with assessing for genetic forms of connective tissue disease are another barrier to a prompt and accurate diagnosis of hEDS [18]. Most (nongenetics) clinicians have limited knowledge and time to accurately diagnose rare genetic diseases. Authors in a recent study reported that only 23% of US physicians surveyed felt very or mostly confident in making a rare disease diagnosis, and more than half of the primary care providers surveyed indicated that insufficient time and knowledge were barriers [24]. This may speak to why, in a 2009 study, most participants with hEDS reported receiving misdiagnoses, sometimes psychological or psychiatric in nature [12]. In another study, individuals with hEDS described feeling belittled and neglected by providers who were skeptical of their symptoms and pain or described experiencing denial of care from providers lacking knowledge of EDS [25]. The limited amount of time that providers typically have with patients, coupled with the increasing complexity of health care and the remarkable phenotypic variability of hEDS, complicates the diagnostic process [10]. As such, decision support systems and web-based tools, which are increasingly being developed to facilitate the provider decision-making process [26], may help providers better recognize symptoms of EDS and offer diagnostic and management recommendations.

### Digital and Diagnostic Tools

Several web-based tools that assist in the diagnostic process have been developed in the genetic or rare disease domain [27-29]. The emerging web-based solutions have generally shown satisfying early results in terms of supporting the diagnosis of rare diseases [26]. In addition, there is evidence that digital tools have high levels of acceptability and satisfaction among patients [30]. An example of a web-based tool intended for medical providers to diagnose another form of connective tissue disease is Marfan Dx [31]. The Marfan Foundation developed the tool using the 2010 Ghent nosology for Marfan syndrome and made it accessible for mobile and desktop users. The aforementioned rare disease diagnostic tools are intended for a medical audience. In the case of hEDS, much of the patient population is extremely active in advocacy and seeking a correct diagnosis, and many have already consulted with providers within the medical community and remain undiagnosed or misdiagnosed. While a provider-facing hEDS checklist and toolkit exist, to the best of our knowledge, no patient-facing app or tool has been previously developed to support their journey to a clinical diagnosis of hEDS.

Patient-facing diagnostic tools have potential to simplify and accelerate the diagnostic journey by raising awareness about the condition and diagnostic process. This could lead to more timely initiation of proper management strategies and improved patient outcomes. Evidence to support this comes from Lee et al [32], who conducted a systematic review on the impact of patient-facing digital tools on patient care. The authors included 70 unique tools, and 84% of the corresponding studies on these tools demonstrated one or more positive outcomes in patient outcome constructs such as knowledge, psychosocial well-being, behavior changes, management changes, family communication, decision-making, or engagement. The authors also found that the digital tools enhanced provider workflow and efficiency and reduced time needed with patients [32]. Given that the hEDS community is very active, a tool for raising awareness among providers may be especially useful. In addition, the diagnostic tool may help prevent false diagnoses; there can be stigmatization that occurs with chronic diseases, and false diagnoses can impose unnecessary restrictions and impact quality of life [33].

### Data Security

The robust use of electronic health records, patient portals, and health-related websites has dramatically increased the amount and types of resources available to the public regarding their health. However, use of such digital tools comes with data security risks, and many health care applications do not meet privacy and security requirements [34]. Given patients’ privacy and security concerns with transmitting medical information [35], building a tool that does not transmit protected health information while still offering the potential to support accurate diagnosis and communication with clinicians could reduce worry surrounding data privacy. An example of an approach to preserve data privacy is to build tools where no protected health information is collected or transmitted and all entered data remain on-device in the form of structured data. In such a scenario, power is given to the patient to protect or share their data by showing or printing information from the application on their device at their discretion.

### Design Thinking and Human-Centered Design

Human-centered design (HCD) and design thinking are methods of innovation that focus on the needs of the human being, or the user or patient, to develop solutions to problems. HCD and design thinking use a variety of tools and methods to understand the problem being experienced by the person or population; identify possible solutions to those problems; and iteratively prototype, test, and refine those solutions [36]. These methods have been used to design and implement digital tools and resources in many health care settings [37-40]. To equip patients with an improved diagnostic pathway for hEDS when a medical genetics provider is inaccessible, we used HCD and design thinking to create a prototype of a web-based diagnostic tool harnessing the current diagnostic criteria. It allows individuals to apply the 2017 diagnostic criteria to their own symptoms and share a summary sheet of their responses with their medical providers. Before developing the tool into a mobile health (mHealth) app, we wanted to understand whether a diagnostic tool prototype aimed at assisting with diagnosing hEDS and facilitating communication between the patients and providers would be needed, acceptable, and useful and hold value to members of the EDS community. A prototype of the diagnostic tool was presented to individuals with a diagnosis of hEDS to answer these questions.

### Frameworks for Evaluating Health Apps

The widespread use of smartphones has led to considerable growth in the development of mHealth apps. Consequently, several mHealth app evaluation frameworks have emerged to help patients and clinicians choose the most suitable and reliable app for their intended use [41]. Using a framework to evaluate an mHealth app can assist in understanding what the app can deliver in terms of privacy and security, quality, and safety and on what clinical evidence an app is based [42]. The Mobile App Rating Scale (MARS) [43] is available for general use and is viewed as a reliable, easy-to-use framework [44]. The MARS contains 23 items across 4 dimensions (engagement, functionality, esthetics, and quality of information) and an app subjective quality scale. Each item can be ranked from 1 (lowest score) to 5 (highest score). The MARS can be used to calculate scores for each dimension, an app quality mean score, an app subjective quality score, and an app-specific score to assess the “perceived impact of the app on the user’s knowledge, attitudes, intentions to change and likelihood of actual change in the target health behavior” [43]. Applying an app evaluation framework is useful to creators of an app to help them understand whether the given app is addressing the needs and goals for which it was created and highlight specific strengths and weaknesses of the app.

### Research Objective

Individuals with hEDS experience several different physical and somatic symptoms that substantially reduce their health-related quality of life [45]. Improving their diagnostic odyssey has the potential to address symptoms sooner and improve medical management. In this study, using a positivist approach, we aimed to understand whether a patient-facing tool intended to support the hEDS diagnostic process would be needed, acceptable, and useful and hold value to members of the hEDS community. We use *tool* throughout this work, which we define as *a diagnostic aid to empower patients and serve as a facilitator to communication and diagnostic assessment with a clinician*. As such, we sought to answer the following research questions: (1) how might an hEDS diagnostic tool help patients communicate information about hEDS to their physicians, influencing the diagnosis of hEDS and affecting patient experiences? (2) What design features do patients with hEDS endorse or recommend in an hEDS diagnostic tool? and (3) How might the usability and utility of the hEDS diagnostic tool be improved across the 4 dimensions of the MARS? We hypothesized that members of the hEDS community would be accepting of a patient-facing tool that could empower them on their diagnostic journey and that they could offer insightful suggestions for optimizing such a tool.

## Methods

### Participants and Recruitment

Participants were recruited through two avenues: (1) private Facebook groups and (2) a clinical database of patients referred and evaluated for various pediatric and adult-onset genetics conditions, including hEDS, maintained at Geisinger Medical Center Department of Genetics and Genomics. This purposive sampling method was used to capture more diversity and include individuals both inside and outside the Geisinger service area. Geisinger is a large, integrated community-based health system that serves central, south central, and northeast Pennsylvania with robust medical and research genetics programs. It serves a large rural and stable patient population [46].

A social media post advertising the study opportunity was posted by a study team member (JG) on the EDS Extension for Community Healthcare Outcomes Health Advocacy Program private Facebook group and the Postural Orthostatic Tachycardia Syndrome or Ehlers-Danlos or Chiari Support Group for Central PA private Facebook group. In total, 2 postings were made on each Facebook group spaced approximately 3 weeks apart. The post provided the email address of JG so that anyone who viewed the advertisement and was interested could receive additional information. A total of 10 individuals who viewed the Facebook post requested additional study information. In addition, participants were identified for the study through Geisinger’s Department of Genetics and Genomics clinical database. A patient list was exported from the database of patients seen in the clinic between September 2019 and September 2021 who were evaluated for hEDS. Patients were excluded from the research if, after chart review, they did not meet the criteria for hEDS following a clinical genetics evaluation. Eligible patients were contacted by study personnel up to 3 times either by telephone or through a patient message on the Geisinger patient portal during a 3-week recruitment period. A telephone script or advertisement were used to introduce the study. Those interested in learning more were sent additional information through encrypted email or the secure patient portal. A total of 12 patients from Geisinger expressed interest and requested additional study information. All participants were required to have a clinical diagnosis of hEDS, be aged ≥18 years, and read and speak fluent English. Study staff privately emailed information about the hEDS diagnostic prototype tool, the goals of the study, a demographic questionnaire, and a questionnaire asking about the diagnostic experience to those who enrolled in the study. Participants were offered 3 focus group times; 2 were offered in the evening and 1 was offered at midday. Recruitment ended after holding 2 focus groups, when theoretical sufficiency was achieved and the authors thought that there was enough breadth and depth of participant data to effectively address the research objectives [47].

### Ethical Considerations

The study was waived as human participant research by both the Geisinger and Sarah Lawrence College institutional review boards (IRBs). All procedures followed were in accordance with the ethical standards of the responsible committee on human experimentation (institutional and national) and with the Helsinki Declaration of 1975 as revised in 2000. Informed consent was obtained from participants using a written consent letter approved by the IRB (Geisinger IRB 2021-0394), which specified that return of the demographic questionnaire implied consent. The research protocol and all study materials, scripts, and advertisements were reviewed by the Geisinger IRB and determined to meet criteria for exemption as defined under category 2 in the US Department of Health and Human Services regulations for the protection of human participants in research. As such, no formal informed consent process was required. However, all participants were provided with an information form regarding the research and were asked to indicate whether they understood that no identifying or contact information would be shared outside of the research team, that participation was voluntary, and that all research data would be kept anonymous and private. No compensation was offered to participants. All participants were asked to agree to keep the focus group discussions private and not share what was discussed after leaving the focus group. To protect the privacy of research participants, all study-related data were deidentified and assigned a unique research identifier, and all survey responses were anonymous.

### Qualitative Data Collection

The focus groups were conducted by 3 or 4 members of the study team, with one member facilitating the internet-based virtual focus group (VFG), another documenting during a brainstorming exercise, and the remaining study team members taking notes and monitoring the chat. All study team members were female. In total, 2 were genetic counseling graduate students (AK and NL) at the time of the study, one was a population health sciences researcher (KR), and one was a senior research genetic counselor (JG) with a clinical interest in EDS and a history of helping establish an hEDS specialty genetics clinic at her workplace. The latter 2 team members had extensive previous experience with qualitative research and trained the student members of the research team. Study team members had no previous relationship with the participants before this study.

We conducted 2 VFGs using the Microsoft Teams (Microsoft Corp) videoconference platform. To ensure privacy, a link was sent to participants individually, and participants were asked to join from a private area. Study team members participated from a private room in their homes. Participants were admitted from the Microsoft Teams waiting room into the VFG, were asked to share only first names, and had the option to have their web camera on or off. A total of 8 participants attended the first VFG, which lasted 1 hour and 28 minutes. A total of 62% (5/8) of the participants opted to have their camera on during this focus group session. In total, 7 participants attended the second VFG, which lasted 1 hour and 32 minutes. Of these 7 participants, 4 (57%) opted to have their camera on during this session. One participant had audiovisual complications and used the chat feature to share all feedback.

At the start of the VFG, ground rules were reviewed (eg, privacy of the discussion, respect for fellow attendees, and turn taking or participation), and a member of the study team announced that recording of the VFG would begin (using the Microsoft Teams recording feature). Participants were asked to share their motivation to take part in the study. A script akin to an interview guide developed by the research team was used to guide discussion on aspects of the usability, usefulness, and acceptability of the tool and began with a demonstration of the tool via the screen sharing feature. The VFG leader (JG) presented a hypothetical individual with some symptoms of hEDS and walked the participants through how the individual answered the questions within the tool by showing wireframes of the tool prototype.

After the demonstration, the Rose, Thorn, Bud design thinking method was used to assess participant reactions to the tool [48]. Participants shared “roses” (positives about the tool prototype or what worked well), “thorns” (negatives about the prototype or what needed improvement), and “buds” (areas for improvement or ideas yet to be explored). Participants were given 5 minutes for each part of the 3-step rose, thorn, or bud activity to brainstorm and write down their responses. Subsequently, participants were asked to volunteer what they documented during the brainstorming activity. A member of the study team (KMR) used a web-based white board (Miro board) with virtual sticky notes to record, color code, and sort “roses” (pink), “thorns” (yellow), and “buds” (green), which were visible to participants. Items were sorted into rough categories, and member checking occurred to ensure correct understanding of the responses.

In addition to discussion, participants used the chat feature and were invited to include additional thoughts in a postinterview survey via an anonymous web-based form. Verbatim transcripts were created from the Microsoft Teams recording. Transcripts, chat logs, and postinterview surveys from VFGs were reviewed, deidentified, and given a study ID. Transcripts and surveys were reviewed for additional roses, thorns, and buds and other remarks that were missed during the VFGs. These new ideas were also color coded based on “rose, thorn, bud” status to stay organized and added as sticky notes to the Miro board. Notes and focus group recordings were stored on Geisinger’s secure Microsoft Teams portal.

### Quantitative Data Collection

**Figure 1:**
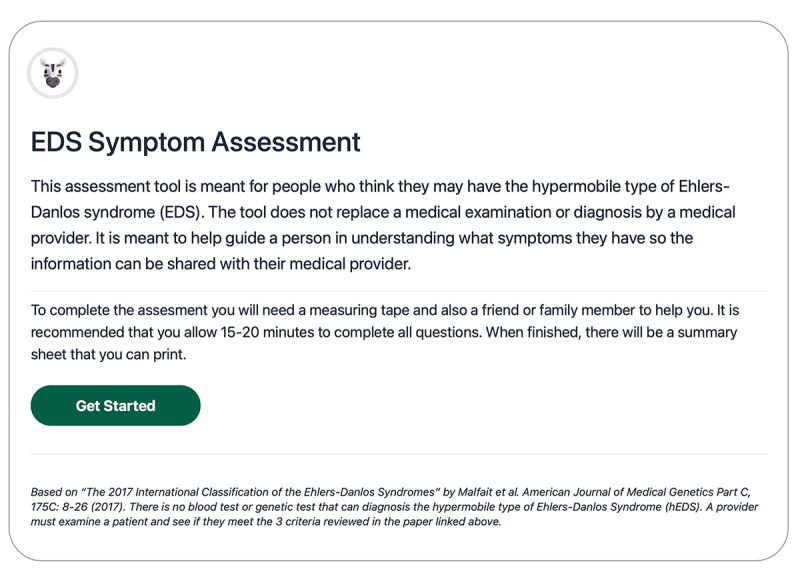
The landing page for the prototype of a patient-facing digital tool to facilitate diagnosis of hypermobile Ehlers-Danlos syndrome (hEDS) is shown in this figure. It includes a disclaimer, the expected time needed to complete the tool, what will be needed to complete the tool, who the tool is intended for, and what the tool will produce.

Participants who attended the VFGs were privately recontacted via email and invited to complete the MARS. Those who expressed interest were emailed a PDF of the MARS, a PDF document that contained all questions used on the diagnostic tool, screenshots of the landing page (Figure 1), a list of the questions in chatbot format, and an example summary sheet from the tool. A total of 2 participants did not recall enough detail about the tool, and therefore, a link to the video demonstration of the tool was also shared. Participants were asked to select the appropriate response for each item on the MARS and return the PDF via email; scoring was completed by the study team. Responses to the MARS were confidential, with no identifiers linking responses to participants.

### Qualitative and Quantitative Data Analysis

Using a phenomenological approach, data analysis involved qualitative content analysis using affinity mapping to create a visual codebook used to code the transcripts. Content analysis allowed for reduction of concepts into key categories [49] through affinity mapping. Affinity diagramming or mapping, also known as the KJ technique, was developed in the 1960s by anthropologist Jiro Kawakita [50] and was well suited for our research to understand patterns of thinking and visualize the qualitative data.

**Figure 2:**
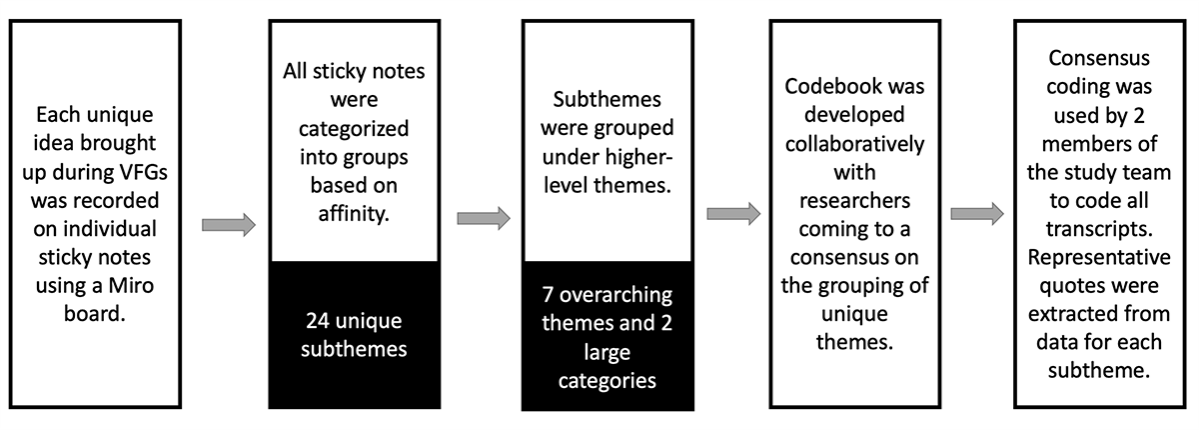
The flow of the affinity mapping process that was used in this study starting from ideas shared during focus groups and ending with qualitative coding of focus group transcripts. VFG: virtual focus group.

Note taking and data clustering into categories based on affinity occurred in two steps: (1) in real time as a member of our research team (KMR) typed web-based sticky notes during the VFGs and (2) after the VFGs, when brainstorming by writing down ideas extrapolated from transcripts, recordings, chat history, and postinterview surveys on web-based sticky notes. Next, the study team met to review the notes from the Miro board and discussed which notes shared similar themes and should be grouped together. Ongoing discussion with team members was vital to clarify and talk through conflicts regarding how the notes should be grouped and how to name the themes and subthemes. As 2 authors had personal experience with (potential) EDS diagnoses within their families and felt that patient education needs to be enhanced, reflexivity was practiced ensuring that the interpretation of the data remained objective and true to the experiences and shared thoughts of the participants [51]. After several iterations, consensus was achieved, and the notes were sorted into the final categories, which were then sorted into overarching themes. This process of data organization facilitated the identification of codes that were used to code the transcripts, chat, and postinterview surveys. Transcripts were coded through consensus by a subset of the study team (NL and AK), and 2 to 4 representative quotes were extracted from the qualitative data for each subtheme. Figure 2 shows the steps that were followed from collecting comments and ideas during the focus groups through qualitative coding of the transcripts.

To visualize what changes and improvements were of highest priority to address for the diagnostic tool, a 2 × 2 prioritization grid was developed in Miro with the x-axis as lift (how much work the change will take) and the y-axis as importance (how many times this was mentioned in the focus group). Finally, the themes and subthemes were mapped to the MARS dimensions of engagement, functionality, esthetics, and information to facilitate understanding of the qualitative data.

Regarding quantitative data, respondents returned the MARS survey, and a member of the study team (JG) scored each one. The MARS total mean score was calculated to ascertain the overall quality of the diagnostic tool, and subscale scores were calculated and compared across participants to identify the strengths and weaknesses of the prototype.

## Results

### Participants

A total of 16 people agreed to participate in the VFGs; 1 (6%) was a no-show. Of the 15 participants, 13 (87%) completed the pre–focus group questionnaires, 13 (87%) self-identified as White and non-Hispanic, 12 (80%) identified as female, and 1 (7%) identified as male. This aligns with studies that note an excess of affected female individuals [52]. Most respondents (9/13, 69%) reported completing a bachelor’s degree or a higher level of education and no difficulty reading health care materials or understanding written information on their condition. An additional 15% (2/13) reported completing some college. Of the 15 total participants, 9 (60%) reported being diagnosed with hEDS by a medical geneticist, and 15 (100%) reported difficulty being diagnosed. Table 1 shows the reported demographics and diagnostic experience of the participants.

**Table 1:** Reported demographics and diagnostic experience of participants with hypermobile Ehlers-Danlos syndrome (hEDS) who took part in focus groups intended to capture their thoughts about and responses to a prototype of an hEDS diagnostic tool (N=15)^a^.

Characteristic and category			Participants, n (%)
**Age range (y)**			
	18-24	2 (13)	
	25-34	2 (13)	
	35-44	4 (27)	
	45-54	3 (20)	
	55-64	2 (13)	
	No answer	2 (13)	
**Years since diagnosis**			
	<1	1 (7)	
	1-4	6 (40)	
	4-10	5 (33)	
	>10	1 (7)	
	No answer	2 (13)	
**Age at diagnosis**			
	Teenage years	1 (7)	
	20s	5 (33)	
	30s	2 (13)	
	40s	5 (33)	
	50s	1 (7)	
	No answer	1 (7)	
**Symptom onset to diagnosis time (y)**			
	1-4	2 (13)	
	5-10	2 (13)	
	>10	9 (60)	
	No answer	2 (13)	
**Relatives with EDS^b^**			
	Yes	5 (33)	
	No	10 (67)	

^a^Participants were recruited from Geisinger Medical Center in central Pennsylvania and from private Facebook groups both within Pennsylvania and across the United States. They were asked to complete a pre–focus group questionnaire to share the characteristics captured in the table.

^b^EDS: Ehlers-Danlos syndrome.

### Qualitative Results

#### Overview

Two main categories of responses to the tool prototype became apparent through the affinity diagramming process: (1) responses related to the user interface and (2) responses related to the application of the tool. These categories each include several themes and subthemes, which were mapped to 1 of the 4 dimensions or MARS subscales (esthetics, functionality, engagement, and information) assessed using the MARS evaluation framework. Table 2 contains the categories, themes, and subthemes identified through affinity mapping and represented in relation to the corresponding MARS dimension.

**Table 2:** Categories, themes, and subthemes identified through affinity mapping and mapped to corresponding Mobile App Rating Scale (MARS) dimensions, which was used as a framework to determine whether the hypermobile Ehlers-Danlos syndrome diagnosis tool prototype addresses the needs and goals for which it was created and highlight specific strengths and weaknesses of the app^a^.

Category and theme			Subthemes		MARS dimension
**User interface**					
	Tool design	Simplicity, visual design, images, and technical design		Esthetics	
	Phrasing	Wording choices and defining medical terminology		Functionality	
	Diversity and inclusion	Gender, race, accessibility, and body diversity		Engagement	
**Application of the tool**					
	Diagnostic process	Defining hypermobility, differential diagnosis, diagnostic criteria, symptoms outside of diagnostic criteria, difficulty identifying symptoms, and measuring arm length		Information	
	Accessing the tool	Where to house the tool and who is using the tool		Functionality	
	Communication	Easing communication with providers, educating providers, and past experience with providers		Functionality	
	Results	Ending summary sheet, possible reactions to results, and diagnostic utility		Information	

^a^Focus groups were held to assess a prototype of a patient-facing diagnostic tool to facilitate improved diagnosis of hypermobile Ehlers-Danlos syndrome. Using the affinity diagramming process to guide qualitative content analysis of focus group interview content, 2 overarching categories of participant responses were evident, along with several themes and subthemes.

#### MARS Dimension: Esthetics

The MARS defines esthetics as “graphic design, overall visual appeal, color scheme, and stylistic consistency” [43]. The “Tool design” theme and 3 subthemes (simplicity, visual design, and images) were mapped to this dimension, as shown in Table 3 with corresponding quotes.

**Table 3:** “Tool design” subthemes, summary of participant comments, and specific quotes mapping to the esthetics” dimension on the Mobile App Rating Scale (MARS)^a^.

Tool design subtheme	Summary	Quote
Simplicity	Strength of tool design, easy to walk through, and “chatbot” style of the tool with questions posed one at a time simplifies flow	“I think it’s very user friendly in that respect. You’re not throwing all of the questions in someone’s face all at once, so you have to answer one question [at a time]. Then it brings you to the next question. I think that that can be really helpful for anybody, but I’m even thinking about people who are more prone to sensory overload, you know, neurodivergent parts of our community? Just having everything all in your face at once can be extremely overwhelming, and so I think that this has a nice flow and it’s certainly easy to use, so I think that’s really great.”
Visual design	Opinions split on design; some thought the colors and icons were positive, others did not	“The colors that you used in [the tool] were kind of drab. I feel like [you could use] different color tones like use different colors. Because I don’t want to get depressed as I’m going through this [because] it may end up that I have bad news.”
Images	Including images was helpful, but some could be improved or added	“What I always thought was normal, and I’ve recently learned isn’t necessarily everyone’s normal, so that was helpful for me to have photos when I was learning about it.”

^a^Focus groups conducted with individuals clinically diagnosed with hypermobile Ehlers-Danlos syndrome (hEDS) were held to assess a prototype of a patient-facing diagnostic tool intended to facilitate improved diagnosis of hEDS. Using the affinity diagramming process to guide qualitative content analysis of focus group interview data, several themes and subthemes were identified. The MARS was used as a framework to map themes, and subthemes, determine whether the hEDS app prototype addresses the needs and goals for which it was created, and highlight specific strengths and weaknesses of the app.

#### MARS Dimension: Functionality

The MARS defines functionality as “app functioning, easy to learn, navigation, flow logics and gestural design of app” [43]. In total, 3 themes (phrasing, accessing the tool, and communication) and their corresponding subthemes (Tables 4 and 5) were mapped to this dimension. In addition, 1 subtheme (technical design) from the “Tool design” theme mapped best to the functionality dimension of the MARS.

**Table 4:** “Phrasing” subthemes, summary of participant comments, and specific quotes mapping to the functionality dimension on the Mobile App Rating Scale (MARS)^a^.

Phrasing subtheme	Summary	Quote
Word choices	Some wording was unclear and closely resembled criteria meant for a medical audience	“I would formulate [the question about stretch marks] differently because I had a lot of them when I was a teenager and I was only slightly overweight...The problem is I would maybe say, ‘more than peers in a similar situation’ or something like that because when I was in my 20s, my best friend had twins and I had more stretch marks than she did with just being slightly overweight.”
Defining medical terminology	Understanding medical terminology is important for filling out the tool correctly	“[I recommend] simplifying the language a little bit so that people that are new to this or don’t really know the medical terminology for things can navigate and assess themselves in the most accurate way possible. That's the only way you survive in this world, as a chronic illness patient, is by learning.”
Spelling	2 typos were identified	“on the word cut, like if you’re using a knife...that was just a little typo.”

^a^Focus groups conducted with individuals clinically diagnosed with hypermobile Ehlers-Danlos syndrome (hEDS) were held to assess a prototype of a patient-facing diagnostic tool intended to facilitate improved diagnosis of hEDS. Using the affinity diagramming process to guide qualitative content analysis of focus group interview data, several themes and subthemes were identified. The MARS was used as a framework to map themes and subthemes determine whether the hEDS app prototype addresses the needs and goals for which it was created, and highlight specific strengths and weaknesses of the app.

**Table 5:** “Communication with providers” subthemes, summary of participant comments, and specific quotes mapping to the functionality dimension on the Mobile App Rating Scale (MARS)^a^.

Communication with providers subtheme	Summary	Quote
Easing communication with providers	The tool could serve as a helpful bridge between patients and physicians because patients can get a better sense of what to talk about with a physician and it can help patients and physicians better identify EDS^b^ symptoms to discuss	“A lot of times when I was first being diagnosed...one, I wasn’t feeling well, and two, the mixture between not feeling well and the nerves would make me really brain foggy. So I’ve learned to have to write everything down so that when I go [to the doctor] I can say, ‘this is what’s going on.’ But even when I just verbally explain it to doctors, I feel like because we have so many seemingly unconnected symptoms, it becomes overwhelming for them. To have something that you can print out and then hand to them and they can just look over it instead of having to verbally tell them and keep track of everything simplifies that whole process and is just way less confusing.”
Educating providers	The tool could help educate providers about hEDS^c^ and the diagnostic process	“Without more EDS awareness both in the public and health professionals, people won’t know about the tool, but with more awareness and the tool, it will help a lot.”
Past experience with providers	Participants had long diagnostic odysseys and struggled to get physicians to believe them about their medical issues	“I’ve actually watched people get injured in front of me by a doctor forcing that stuff (demonstrating a precise Beighton score), saying, ‘Oh well, you’re not that bendy and she’s like OK, watch here’ and she injured herself.”

^a^Focus groups conducted with individuals clinically diagnosed with hypermobile Ehlers-Danlos syndrome were held to assess a prototype of a patient-facing diagnostic tool intended to facilitate improved diagnosis of hypermobile Ehlers-Danlos syndrome. Using the affinity diagramming process to guide qualitative content analysis of focus group interview data, several themes and subthemes were identified. The MARS was used as a framework to map themes and subthemes, determine whether the hypermobile Ehlers-Danlos syndrome app prototype addresses the needs and goals for which it was created, and highlight specific strengths and weaknesses of the app.

^b^EDS: Ehlers-Danlos syndrome.

^c^hEDS: hypermobile Ehlers-Danlos syndrome.

Regarding the “Accessing the tool” theme, 2 subthemes were identified that mapped to the functionality dimension on the MARS: *where to house the tool* and *who is using the tool*. Participants expressed that the tool should be accessible both in person and on the web and advertised via social media and in certain types of physician offices, as the internet is not available to all. One participant remarked, “I was back and forth to orthopedists for years just being misdiagnosed, year after year after year. If I had stumbled on a leaflet in a waiting room, I might be able to grab it and bring it to the doctor and say, tell me more about this and who I can go to to learn more, because I have all of these things. It is something that maybe could have helped me be diagnosed back in the 90s instead of 2018.” When addressing who is using the tool, there were varying opinions with some thinking the tool is best accessed by physicians. Others thought the tool should be used by patients. For example, “I think the patient should fill out the information. Patient empowerment is important, and the patient knows their body best. I’d bring the results to my doctor.” Still other participants thought the tool should be available for both patient and physician use. Regardless, participants felt that the intended audience of the tool should be clearly labeled.

The *technical design* subtheme of the “Tool design” theme mapped to the functionality dimension on the MARS. To summarize qualitative content related to this subtheme, participants offered suggestions to add functionality to the tool, such as skipping an item or adding an “N/A” option. One participant commented, “Maybe adding in a back feature so that if you remember something later, you can just click backwards and then change it” would be useful.

#### MARS Dimension: Engagement

The MARS defines engagement as “fun, interesting, customizable, interactive, well-targeted to the audience” [43]. Several subthemes mapping to this dimension were identified from affinity diagramming that were all felt to relate to the theme of diversity and inclusion. These encompassed comments on gender, race, accessibility, and body diversity, as shown in Table 6.

**Table 6:** “Diversity and inclusion” subthemes, summary of participant comments, and specific quotes mapping to the engagement” dimension on the Mobile App Rating Scale (MARS)^a^.

Diversity and inclusion subtheme	Summary	Quote
Gender	Use of language and terminology inclusive of transgender and intersex individuals	“I’m not an expert on the correct way to address gender, but I think the word gender assignment is the correct way to have people say I was a male at birth. I was a female at birth. Our population tends to have a lot on the LGBTQ spectrum, so whatever phrase is correct.”
Race	Include more images of bodies with different shades of skin to avoid stereotyping and assist in recognizing skin findings in those with darker skin	“I think that just having more diversification in the photographs that people see because there’s this generalization or stereotype that doctors have in their head...it’s stemming from the fact that all of the pictures that they’re seeing in their publications or when they go to conferences and workshops and things like that they’re seeing, generally speaking, petite young white women. EDS is not specific to that...you know how is somebody who’s black or brown supposed to know what atrophic scarring looks like on their skin when every picture that’s published of atrophic scarring with respect to EDS is of white skin?”
Accessibility	Including technology to make the tool accessible to most	“Regarding disability, I don’t know how alt text works, but that tends to be a norm to provide some alt text for people who may not have the ability to see the visuals.”
Body diversity	Include images showing greater diversity in body size or composition to avoid stereotyping of hEDS^b^ in thin women	“[I wish that] these images weren’t tending toward skeletonized humans. I think only 50% of us are super skinny, the rest of us are of normal weight, many of us are overweight. I think we’re ignoring the overweight population. Is there any way to get some chubbier folks’ images in there?”

^a^Focus groups conducted with individuals clinically diagnosed with hypermobile Ehlers-Danlos syndrome were held to assess a prototype of a patient-facing diagnostic tool intended to facilitate improved diagnosis of hypermobile Ehlers-Danlos syndrome. Using the affinity diagramming process to guide qualitative content analysis of focus group interview data, several themes and subthemes were identified. The MARS was used as a framework to map themes and subthemes, determine whether the hypermobile Ehlers-Danlos syndrome app prototype addresses the needs and goals for which it was created, and highlight specific strengths and weaknesses of the app.

^b^hEDS: hypermobile Ehlers-Danlos syndrome.

#### MARS Dimension: Information

The MARS defines information as “high quality information from a credible source” [43]. Through the affinity diagramming process, 2 themes and multiple subthemes under each (Tables 7 and 8) were found to map to the information dimension. The largest theme that was identified, “Diagnostic process,” encompassed how the diagnosis of hEDS is made and ways in which this tool could be made better in its ability to identify those who should consider being evaluated for hEDS. The “Results” theme emerged later in the categorization process as it became clear that a few of the emergent subthemes related specifically to the results given by the tool but not necessarily to easing communication with providers or the diagnostic process.

**Table 7:** “Diagnostic process” subthemes, summary of participant comments, and specific quotes mapping to the information” dimension on the Mobile App Rating Scale (MARS)^a^.

Diagnostic process subtheme	Summary	Quote
Defining hypermobility	Could be hard to assess hypermobility through the tool’s questions; include items to help distinguish between hEDS^b^ and hypermobility spectrum disorder	“So, I know that there’s a very fine line [between being hypermobile and having Ehlers Danlos], but maybe to help distinguish the difference between the two, because there are people who are hypermobile but do not necessarily have Hypermobility Ehlers Danlos, maybe you can include even more symptoms in there beyond just flexible joints and heart issues.”
Differential diagnosis	Concern about overdiagnosing hEDS or missing other important diagnoses; include items to help rule out other conditions	“I guess what I’m hitting on is diagnostic overshadowing. I want to [make sure] that we don’t end up inadvertently engaging in diagnostic overshadowing with hEDS. Just because a person who’s desperate for answers may have, for instance, Loeys Dietz Syndrome or Marfan or Sticklers or a rare form of EDS. They don’t know all those things, not even all doctors know that there are actually 13 rare types of EDS right now.”
Diagnostic criteria	Some disagreed with the 2017 criteria or wanted the tool to align more precisely with the criteria	“So, I just want to make sure that I don’t pin issues that I have with the 2017 criteria for hEDS as it’s currently stated on this project. But unfortunately, since you’re basing this on those criteria, the issues that I have with those criteria are going to spill over.”
Symptoms outside of the diagnostic criteria	Include common symptoms outside the diagnostic criteria in the tool	“Having a little comment section and even to include other symptoms [would be helpful]. Up until a few years ago, I’ve never even heard of Ehlers Danlos syndrome, and I’ve had everything from chronic migraines to...extremely painful periods. I never knew that was all encompassed into Ehlers Danlos syndrome until after I was diagnosed.”
Difficulty identifying symptoms	It can be hard to identify or compare their symptoms to what is “normal” as what they experienced their whole life is normal to them	“I know one of my dilemmas with how my progression has gone was I always twist my ankle getting off the school bus, so over time I became very cautious, and I didn’t have a lot of these [joint instability] problems because of how careful I was compared to what I would be if I just did whatever a normal person did.”
Measuring arm span	This item may be hard to answer if someone does not have anyone nearby or at home to help them with this measurement	“A couple doctors have shown me how to easily do the wingspan measurement instead of attempting to throw yourself against the wall like Jesus Christ and try and do a measurement of your arms, [which is] just extremely difficult to get accurate. Within 10 seconds you can stand against the door jam, measure how tall you are, make a pencil mark, then immediately reach down, put your fingertips on the floor and reach your arms up...No measuring tape even needed.” [Not clinically recommended]

^a^Focus groups conducted with individuals clinically diagnosed with hypermobile Ehlers-Danlos syndrome were held to assess a prototype of a patient-facing diagnostic tool intended to facilitate improved diagnosis of hypermobile Ehlers-Danlos syndrome. Using the affinity diagramming process to guide qualitative content analysis of focus group interview data, several themes and subthemes were identified. The MARS was used as a framework to map themes, and subthemes, determine whether the hypermobile Ehlers-Danlos syndrome app prototype addresses the needs and goals for which it was created, and highlight specific strengths and weaknesses of the app.

^b^hEDS: hypermobile Ehlers-Danlos syndrome.

**Table 8:** “Results” subthemes, summary of participant comments, and specific quotes mapping to the information dimension on the Mobile App Rating Scale (MARS)^a^.

Results subtheme	Summary	Quote
Ending summary sheet	The tool’s summary sheet was seen as positive as it could be printed and shared; suggestions were given to improve it	“I think [there is a need for] resource links for the patients who do meet the criteria as it can be a big shock, especially if you think of the person who just thought they were a bit clumsy and a bit bendy...and they learn about this new illness they never heard about. I think the reaction might be to not believe it or not do anything about it. They might need to learn more and why it’s important to take it seriously.”
Possible reactions to results	Many felt that receiving a “screen positive” result would feel validating, whereas a “screen negative” result could be disappointing; stressed the need to discuss results with a provider	“I think if I found this tool, I would have been sitting there with my mouth agape that everything got checked off. And so, for me, I would go on a mission to find that medical practitioner who I know would be open minded enough to look at this and help me get to the bottom of it. I found out about my EDS or potential EDS in an online support group on Facebook. That’s off the charts amazeballs in my opinion, but it’s filled with people like us who muddled through the system and hit on all these disparate symptoms. [If I found this tool], I would have felt...validated like why does this one tool have every single symptom I have?”
Diagnostic utility	Participant consensus was that the tool would have been useful to have before their diagnosis; could have saved years on their diagnostic odyssey	“Once I would’ve learned about hEDS, I definitely would’ve used the tool, and it might have saved me a few months...maybe if it had been around the tool would’ve been sent to me much earlier and I would’ve saved years! I’m sure having the tool would’ve made the experience more positive, as I would’ve had something clear to use and show my family and my doctors instead of bringing them different articles they didn’t want to go through, or criteria lists from support websites they didn’t trust.”

^a^Focus groups conducted with individuals clinically diagnosed with hypermobile Ehlers-Danlos syndrome (hEDS) were held to assess a prototype of a patient-facing diagnostic tool intended to facilitate improved diagnosis of hEDS. Using the affinity diagramming process to guide qualitative content analysis of focus group interview data, several themes and subthemes were identified. The MARS was used as a framework to map themes and subthemes, determine whether the hEDS app prototype addresses the needs and goals for which it was created, and highlight specific strengths and weaknesses of the app.

### Quantitative Results (MARS)

Of the 15 VFG participants who were invited to complete the MARS, 3 (20%) returned the rating scale for scoring. The scores for each dimension per participant are shown in Table 9. The subjective quality scores and mean score per participant (Table 10) capture whether the app is worth recommending and stimulates repeated use and assess overall satisfaction with the app. Section F of the MARS is the customizable app-specific rating scale to assess the perceived impact of the app on awareness, knowledge, attitudes, intention to change, help seeking and behavior change. All responses are captured on a Likert scale (1 being the lowest score, 5 being the highest score). The app-specific rating scores are shown in Table 11.

**Table 9:** The scores the participants provided for each dimension of the Mobile App Rating Scale (MARS)—mean scores (could range from 1 to 5) of the engagement, functionality, esthetics, and information quality objective subscales and overall mean app quality score per participant^a^.

MARS dimensions	Participant ID 06	Participant ID 08	Participant ID 13
Engagement subscale^b^, mean (SD 0.2)	3.4	3.6	3.8
Functionality subscale^c^, mean (SD 0.76)	4.5	3.5	5
Esthetics subscale^d^, mean (SD 0.72)	3.6	4	5
Information quality subscale, mean (SD 0.68)	3.8	3.5	4.8
App quality, mean (SD 0.54)	3.82	3.65	4.65

^a^The MARS is a framework available to help determine whether an app addresses the needs and goals for which it was created and highlight specific strengths and weaknesses of the app. Focus groups that included a total of 15 participants were held to provide feedback on a prototype of a diagnostic tool intended to help facilitate accurate and timely diagnosis of hypermobile Ehlers-Danlos syndrome. A total of 20% (3/15) of the participants who attended the focus groups completed the MARS.

^b^In the engagement subscale, question 1 assesses the entertainment value; one participant noted that the app is not intended for this purpose. Question 3 asks about customization; one participant noted that it is not necessary to customize this app for its intended use.

^c^In the functionality subscale, one participant noted that using a live app versus a demonstration of the prototype would have helped better answer the question.

^d^In the esthetics subscale, question 12 assesses visual appeal; one participant responded that the app is not meant to be visually appealing and endorsed a neutral rating.

**Table 10:** App subjective quality scores reported by the 3 participants^a^.

App subjective quality item	Participant ID 06	Participant ID 08	Participant ID 13
Would you recommend this app to people who might benefit from it?	4	4	5
How many times do you think you would use this app in the next 12 months if it was relevant to you?	2^b^	2^b^	3
Would you pay for this app?	1	1	3
What is your overall rating of the app?	4	4	4
Mean score (SD 0.58)	2.75	2.75	3.75

^a^The Mobile App Rating Scale (MARS) is a framework available to help determine whether an app addresses the needs and goals for which it was created and highlight specific strengths and weaknesses of the app. Focus groups that included a total of 15 participants were held to provide feedback on a prototype of a diagnostic tool app intended to help facilitate accurate and timely diagnosis of hypermobile Ehlers-Danlos syndrome. A total of 20% (3/15) of the participants who attended the focus groups completed the MARS. Section E of the MARS is an app subjective quality rating scale, with responses on a Likert scale (1 being the lowest score and 5 being the highest score). The exception is the item that asks whether the respondent would pay for this scale, in which potential answers include *no* (1), *maybe* (3), and *yes* (5).

^b^Additional comments noted by participants: “The App is only intended to be used once.”

**Table 11:** App-specific rating scale scores reported by the 3 participants^a^.

App-specific item	Participant ID 06	Participant ID 13	Participant ID 08
This app is likely to increase awareness of the importance of addressing a thorough and proper assessment for diagnosing hEDS^b^.	4	5	3
The app is likely to increase knowledge and understanding of the process for diagnosing hEDS.	5	5	5
This app is likely to change attitudes toward improving the diagnostic process for hEDS.	3^c^	4	5
The app is likely to increase intentions and motivations to address accurately assessing for the presence or absence of hEDS.	5	4	4
Use of this app is likely to encourage further help seeking for a correct diagnosis for symptoms that may be consistent with hEDS or another similar condition.	5	5	5
Use of this app is likely to increase receiving an accurate diagnosis for symptoms that may be consistent with hEDS.	4	4	3

^a^The Mobile App Rating Scale (MARS) is a framework available to help determine whether an app addresses the needs and goals for which it was created and highlight specific strengths and weaknesses of the app. Focus groups that included a total of 15 participants were held to provide feedback on a prototype of a diagnostic tool app intended to help facilitate accurate and timely diagnosis of hypermobile Ehlers-Danlos syndrome. A total of 20% (3/15) of the participants who attended the focus groups completed the MARS. Section F of the MARS is the customizable app-specific rating scale to assess the perceived impact of the app on awareness, knowledge, attitudes, intention to change, help seeking, and behavior change. All responses are on a Likert scale (1 being the lowest score and 5 being the highest score).

^b^hEDS: hypermobile Ehlers-Danlos syndrome.

^c^Additional comments noted by participants: “Unsure if the App can change the attitude of health care professionals because some don’t believe in hEDS.”

### Motivations to Participate

At the beginning of both focus groups, each participant was invited to share their motivations to take part in the study. Although not part of our assessment of the tool, this yielded rich insights into the hEDS diagnostic experience. Many participants shared difficulties they faced in the process of being diagnosed. Their struggles motivated them to be involved in research to improve the diagnostic process for others and increase awareness. Some participants also mentioned that this study’s purpose aligned with their personal goals and values.

On the topic of personal experience, participants discussed money spent, years to receive a diagnosis, and feeling invalidated by medical professionals. One participant shared that they had spent US $10,000 in a year for their diagnosis. Another shared that they were diagnosed after 20 years, during which they saw ≥200 physicians and spent approximately US $100,000. One mentioned that it took so long to receive a diagnosis that they wanted to help others so that they did not have to experience the same diagnostic odyssey. One participant shared that their diagnosis journey involved a “vicious game of connecting the dots for over 30 years.” Some participants voiced that they were sick of medical gaslighting, for example, being accused of being drug seeking or labeled as crazy, lazy, or stupid by medical professionals. One participant shared that they were adopted; the lack of family history made their diagnostic odyssey extremely frustrating and emotionally exhausting.

Some participants discussed their personal goals and values and how that led them to joining the focus group. One participant shared that they took any opportunity to help the EDS or rare disease communities. Some mentioned that they were active in the EDS and disability communities and wanted to support research. Other participants were passionate about knowing the importance of good data and information in the hEDS community, as well as being involved in hEDS medical education.

Increasing awareness of hEDS is one goal that was shared among participants. Participants mentioned that there needs to be more patient rights, more resources, and more EDS-specific practitioners. One participant shared that it is hard to be diagnosed and find resources to determine whether being diagnosed is worthwhile. Another shared that they were newly diagnosed and they wanted to be able to better inform their physicians about EDS. The need to educate providers about hEDS can feel uncomfortable; improving provider awareness motivated some to participate in this research.

## Discussion

### Qualitative Results

After reviewing transcripts from the VFGs, we were able to identify the strengths of the tool from the perspective of the hEDS patient community and understand the aspects of the tool that need to be readdressed. We learned that the concept of a patient-facing tool held value and utility among the participants and that the simple, chatbot-style format was acceptable and user-friendly. The VFG participants confirmed what is found in the literature regarding long diagnostic odysseys for patients with hEDS. Their stories and experiences clearly support the need for improved communication between individuals with hEDS and health care providers, specifically as they search for a diagnosis. Their responses suggest that this tool has the potential to address this issue, possibly saving users time, money, and energy spent during their diagnostic odysseys and motivating users of the tool to receive an accurate diagnosis. There were several insightful suggestions for areas of improvement.

The shareable ending summary sheet provided by the tool was seen as the most promising piece for facilitating communication between patient and provider during the diagnostic evaluation. Given that some participants were concerned that individuals with other forms of EDS may be misdiagnosed or go undiagnosed after using the diagnostic tool, listing key symptoms (eg, major diagnostic criteria) for other forms of EDS to flag potential differential diagnoses was highly endorsed. Several participants offered suggestions of symptoms to be included in a checklist to expand on both common hEDS comorbidities and on symptoms of other forms of EDS and connective tissue disease. Several participants also suggested including a comment section for adding other symptoms or information to augment communication between patients and providers. As individuals with hEDS often have many seemingly unrelated symptoms, this list could help providers and users alike keep track of symptoms. Participants also endorsed adding resource links to the ending summary sheet, which could help users educate themselves on hEDS. These important suggestions indicate that both empowering patients to communicate with their providers and educating providers about hEDS were seen as equally important for improving the diagnostic process. We envision being able to use these comments to expand what can be entered into a future app to include a section where users can record symptoms, comorbidities, and medications. In addition, current, authoritative resources can be linked within the app. Language regarding the exclusion of differential diagnoses, which was very important within our study cohort, can also be added.

The participants’ responses during the VFGs suggested that there were some concerns about the ability of the tool to accurately flag individuals with hEDS. As the questions in the tool are based on the 2017 diagnostic criteria, some of these concerns might point to a larger issue with the diagnostic criteria themselves. In line with this concern, the Toronto GoodHope EDS Clinic conducted a retrospective cohort study to assess the accuracy of the 2017 diagnostic criteria [53]. Before their study, they noticed that many patients who were highly symptomatic for hEDS were not meeting the 2017 criteria and were left undiagnosed. Their findings, which suggest a need to refine the 2017 hEDS diagnostic criteria to enhance diagnostic accuracy, echo findings from our qualitative analysis. Despite concerns with certain items on the tool and the 2017 criteria, overall, the idea of the tool was seen as useful. Specific questions could be adjusted in the future as the diagnostic criteria for hEDS evolve. However, one of the most frequently mentioned areas of concern for the tool was misattributing symptoms of vascular EDS to hEDS, an error that could result in serious illness or death. To avoid this, participants suggested highlighting information for both the user and provider on the differential diagnoses in the summary sheet and stressing the importance of ruling out vascular EDS after a “screen positive result.” As participants pointed out, adding more information about the differential diagnosis could also be important for those who “screen negative” and may not know where to go next. Including information on differential diagnoses can be easily incorporated into a future application. Another suggestion that could improve the tool’s ability to facilitate accurate diagnoses was including a larger family history section. Although it would not factor into whether someone “screens positive” using the tool, it would give providers seeing the summary sheet a fuller picture of the family’s overall health, potentially helping with a differential diagnosis for those who screen negative. Some participants suggested making the intended audience for this tool clearer, as well as what it can or cannot do. Plans for an app include having clearly labeled provider and patient sections. Interestingly, misuse of the tool was not a concern that was specifically brought up in either focus group.

**Figure 3:**
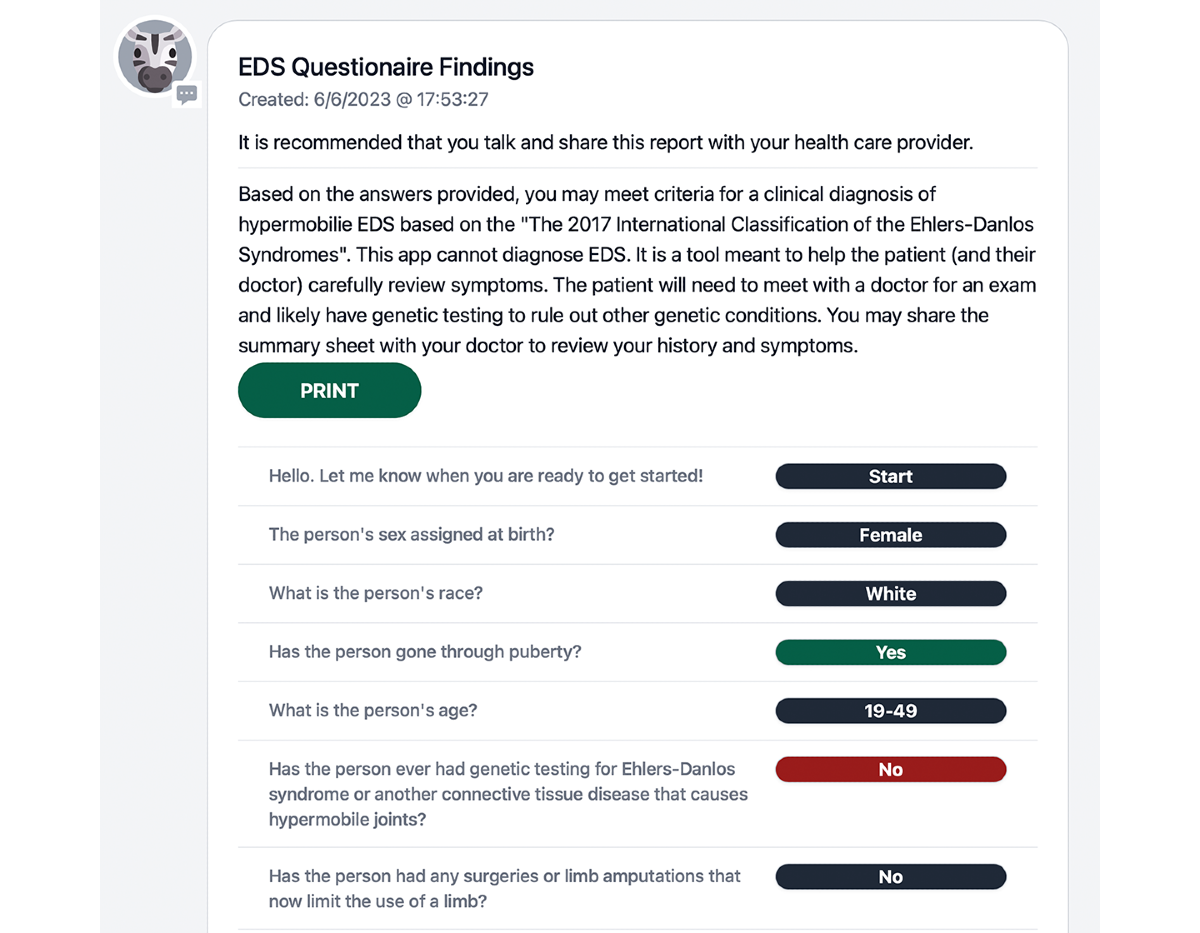
This is an example of the first page of a multipage printable summary sheet that would be produced from answering the questions on the app. Users can opt to share it with their medical provider to facilitate discussion about their symptoms in relation to the diagnostic criteria for hypermobile Ehlers-Danlos syndrome (EDS).

The depth and diversity of suggestions on the user interface imply that this is an important component of the user’s experience with the tool. Word choice was a frequently mentioned area for improvement. Although the word choice for the questions on the tool was intended to be consistent with the 2017 diagnostic criteria yet adapted to be patient facing, participants had many suggestions for areas where the word choices were confusing and medical jargon could be clarified, making the tool more accessible to the general population. Recommendations for text-to-speech capability and the inclusion of alternate text describing images were also made. The language used regarding sex and gender was suggested to be updated and clarified. Asking for “sex assigned at birth” rather than “sex at birth” and including an option for “other” was one suggestion from participants to improve the precision and the inclusivity of the tool. This wording, in addition to rephrasing some other items, has already been incorporated (Figure 3).

Participants offered many suggestions for improvements to the images used in the tool. Showing degrees of variability and images comparing someone with hEDS versus someone without could add further clarification. Including images of more diverse bodies could also help ensure that users of all skin tones and body types could easily recognize manifestations of hEDS on their own bodies. This would ultimately improve the clarity and inclusivity of the tool and combat stereotypes of what individuals with hEDS look like.

Another aspect of the user interface that drew comments involved the design and functionality of the tool. Although the chatbot format was seen as a positive attribute, it limits functionality. The participants suggested that a back button or skip logic could make the tool easier to use. Other aspects of design, such as colors and icons, were more controversial, and it is unlikely that any design choice will please all users. User feedback has been considered, and an app with more functionality and flexibility is planned.

The participants’ responses on where to house the tool suggest that it would be best to have the tool available in many places both on the internet and in person. Advertising the tool via pamphlets or brochures in certain physicians’ offices was suggested during the VFGs. Housing the tool solely on the web, on the Ehlers-Danlos Society website specifically, for example, may limit the number of people who could benefit from it. As participants pointed out, many have limited access to the internet or spend little time on the web. For those who do spend time on the web, they might only find the tool on the EDS website if they are already familiar with EDS, limiting its usefulness. To ensure that the tool is widely accessible, participants suggested workshopping good search engine optimization terms and making the tool usable on both mobile and desktop. They also suggested having professional or medical society backing to add more validity and help ensure that providers would be receptive to users’ results.

### Quantitative Results (MARS)

Scoring the returned MARS surveys indicated that the EDS diagnostic tool prototype was moderately highly to highly rated among our respondents, which echoed the qualitative analysis findings. App quality scores showed that the tool was easy to navigate, functioned well, and was moderately to highly visually appealing. This is consistent with qualitative data gathered from the VFGs, where most participants liked the easy flow and chatbot style of the tool and felt that the design was pleasing. On the basis of the items assessing engagement (eg, interactivity, interest, target group, customization, and entertainment), respondents were moderately engaged with the app. One participant noted within the engagement subscale that the app is not intended to be used for entertainment, and they rated it accordingly. Another respondent noted within this subscale that it is not necessary to customize this app for its intended use. As the diagnostic tool is not meant to entertain or be interactive, a moderate engagement score is not surprising. There were differences among survey respondents on the subscale assessing information quality, making it challenging to draw conclusions from the small cohort of respondents. This, in part, stemmed from a MARS item assessing where the app was developed (eg, an academic center or a company); this is not clear on the prototype, nor was it discussed in the VFGs. A future version of the tool would need to clearly indicate the developer. Another question with divergent responses regarded whether the content of the app was well written. While 67% (2/3) of the respondents highly endorsed this item, 33% (1/3) of the respondents indicated that they did not like how some questions were addressed to the patient, whereas other questions were addressed to a provider. Although most questions in the EDS diagnostic tool were structured to be patient facing, the phrasing of a few items was kept as provider facing to be consistent with the published questionnaire by Hakim and Graham [54] assessing hypermobility. Consistent phrasing of questions in a future version may reduce confusion. In general, information quality was perceived as moderate to high.

The MARS app subjective quality scores indicated that the tool had a high satisfaction rating, and respondents valued the tool enough to warrant recommending it to others. This reflects discussion during the IFGs where participants indicated they would be likely to recommend the tool to a friend with signs of EDS. Within the subjective quality subscale, when asked whether respondents would pay for the app, 67% (2/3) responded “no,” whereas 33% (1/3) responded “maybe.” On the item assessing repeated use of the app, a lower score was endorsed by most respondents, with 67% (2/3) noting that the tool is not intended for repeated use. The app subjective quality scores indicated that the respondents feel that others could benefit from the app but do not feel that they would pay for the app. This may reflect that the app is intended to be a 1-time use experience, and consumers may not feel that this warrants payment. In addition, during the VFGs, we discussed that the app was intended to be available on a website, such as the Ehlers-Danlos Society site, which may have led respondents to believe or feel that the tool should be free.

The app-specific rating scale scores showed overwhelmingly that the respondents felt that the EDS diagnostic tool would likely increase the knowledge surrounding the diagnostic process for hEDS. Scores indicated that the tool would encourage users to continue to seek help to obtain a correct diagnosis. Most respondents felt that use of the tool would increase the likelihood of receiving an accurate diagnosis and would increase awareness of the importance of properly assessing for hEDS. Interestingly, when asked to rate whether the tool is likely to change attitudes toward improving the diagnostic process for hEDS, 67% (2/3) of the respondents highly endorsed this item, whereas 33% (1/3) noted that they were uncertain of whether a diagnostic tool or app can change the attitude of health care providers because some simply “don’t believe in” hEDS.

### Limitations

Participants were recruited to this study because they had diagnoses of hEDS, although the sample is not representative of all individuals diagnosed with hEDS, especially given our homogeneous sample. This may affect how transferrable their responses are to a wider audience. Most participants also reported feeling generally comfortable reading health care materials, and some were part of advocacy groups or reported being highly involved in work surrounding hEDS. As such, our sample may be more informed on hEDS than the average user of the diagnostic tool and more inclined toward using a digital tool. Further testing is needed with a general-population user group. Focus groups are a group-based technique subject to group bias effect [55], which brings up the possibility that the participants may not express their honest and personal opinions on the topic. Another limitation is technical difficulties encountered by one participant, limiting their participation to text only via the chat feature.

Regarding the MARS, technically, the scale is not intended for use on a static prototype, although we felt that using the MARS as a basis to improve the quality of a future EDS diagnostic tool was acceptable. In addition, because survey respondents were not trained on how to use the MARS, it is possible that respondents did not accurately or consistently rate items on the MARS. Finally, it could have been beneficial to have additional respondents to the MARS survey to ensure that the needs and goals for which the tool was created were on target and highlight additional strengths and weaknesses. Despite this, having qualitative data that assessed user experience from 2 VFGs likely allowed us to capture most themes surrounding the utility, feasibility, acceptability, and need for a diagnostic hEDS tool. According to a 2017 study, 80% of themes are discoverable within 2 to 3 focus groups, whereas 90% are discoverable within 3 to 6 focus groups [56]. When assessing user experience, usability tests including 5 users are considered ideal, whereas including 15 users typically allows for the discovery of all usability problems [57,58].

### Research Recommendations

Research is underway to identify the genetic etiology of hEDS. In the meantime, this study could lead to further research on current EDS diagnostic criteria and the need to enhance the diagnostic process. On a larger scale, further research is needed into barriers to care for individuals with hEDS and the impacts of this on quality of life. Finally, it is important to investigate whether patient-facing digital tools could be beneficial for individuals seeking diagnoses for other genetic or medical conditions that rely on clinical diagnoses and whether access to a diagnostic tool could improve accurate application of diagnostic criteria. For example, fibromyalgia may be an interesting candidate for a similar tool given the lack of objective markers and availability of diagnostic criteria [59]. Studies have shown that patient-facing digital tools can be used successfully to support components of pretest genetic counseling and education and can enhance workflow and efficiency for clinicians [32]. There is the potential for scalability and adaptability of the digital tool described in this paper for conditions (genetic and medical) that currently have published diagnostic criteria or guidelines that can be put in digital format.

### Future Directions

Since the conclusion of this research, the prototype of the EDS diagnosis tool has been presented to the Ehlers-Danlos Society. Given the shared goal of improving the diagnosis of those with EDS, this has led to a collaboration. A project envisioned by the Ehlers-Danlos Society has been undertaken by some of the world’s leading experts on EDS to generate and publish the diagnostic pathways for the EDSs. An mHealth app similar but more extensive than the prototype used in this research is in the process of being developed to detail these diagnostic pathways in electronic form and will include both patient- and provider-facing sections. We are anticipating using React, which is an extensible, mature JavaScript, to build the user interface. In alignment with the desire voiced by participants in the VFGs to record their potential symptoms of EDS, the web-based tool will include a patient-centered section with a symptom diary, a medication reminder feature, and a social platform to allow for connections among users.

### Conclusions

Assessing the use of this diagnostic tool from the perspective of individuals diagnosed with hEDS has highlighted how it could be useful for individuals searching for an explanation for their potential hEDS-related symptoms. Suggestions and improvements were offered to optimize the tool before public release. Participants indicated that the tool may help individuals be diagnosed more easily, save time and money, help educate providers, and overall improve the diagnostic odyssey of hEDS. While they offered detailed suggestions for improvement, their overall feedback was positive and enthusiastic about the concept of the tool in general and expressed their desire to see it become available to patients. Recommended changes include altering the technical and visual design, incorporating more diverse language and images, and simplifying wording and medical terminology while consistently being patient facing. These suggestions will aid the process of finalizing a diagnostic tool and may, ultimately, help facilitate a diagnosis for individuals with hEDS.

## Abbreviations

EDS: Ehlers-Danlos syndrome
HCD: human-centered design
hEDS: hypermobile Ehlers-Danlos syndrome
IRB: institutional review board
MARS: Mobile App Rating Scale
mHealth: mobile health
VFG: virtual focus group

## Data Availability

The data sets generated during and analyzed during this study are available from the corresponding author on reasonable request.
